# Outcomes of Full-Right-Full-Left Split Liver Transplantation in Adults in the USA: A Propensity-Score Matched Analysis

**Published:** 2016-05-01

**Authors:** A. Zimmerman, J. M. Flahive, M. Hertl, A. B. Cosimi, R. F. Saidi

**Affiliations:** 1 *Division of Organ Transplantation, Department of Surgery, Alpert Medical School of Brown University, Providence, RI, USA*; 2 *Center for Outcomes Research, Department of Surgery, University of Massachusetts Medical School, Worcester, MA, USA*; 3 *Division of Transplantation, Department of Surgery, Rush University Medical Center, Chicago, IL, USA*; 4 *Transplantation Unit, Department of Surgery, Massachusetts General Hospital, Harvard Medical School, Boston, MA, USA*

**Keywords:** Liver transplantation, Waiting Lists, Mortality, Allograft survival, Patient survival, Survival analysis

## Abstract

**Background:**

Splitting a liver for utilization in adult/pediatric recipients has been shown to decrease mortality on the wait list without increasing the overall risk of long-term graft failure compared to a whole graft. However, splitting a single donor organ for two adult recipients, full-right-full-left split liver transplantation (FRFLSLT), to overcome organ shortage is still considered controversial.

**Objective:**

This study assessed the outcome of FRFLSLT comparing full-right (FR) and full-left (FL) with whole liver (WL) allografts in adults (1998–2010) using UNOS standard transplant analysis and research (STAR) file.

Methods: Unadjusted allograft and patient survival were estimated using Kaplan-Meier survival curves. Adjusted analyses of survival were conducted controlling for propensity for WL allograft.

**Results:**

There were 83,313 cases of WL, 651 FR and 117 FL. Significant differences were evident in the unadjusted cohort between recipients who received FR and FL including donor, cold ischemic time, and days on transplant waiting list. Use of FL allograft resulted in a trend toward lower graft and patient survival compared to WL and FR, which was not statistically significant (p=0.07). In the matched cohort, FL hemiliver allograft had no detrimental effect on the allograft or patient survival after split liver transplantation when compared to FR and WL.

**Conclusion:**

After adjusting for donor and recipient characteristics, there was no difference in allograft or patient survival with the use of FL, FR, or WL after liver transplantation in adults. FRFLSLT is a valuable and safe option to expand the donor pool.

## INTRODUCTION

The number of liver transplantations being performed each year is ultimately limited by graft availability. Despite the increasing demand for orthotopic liver transplantation (OLT), supply of deceased donor organs has persisted as the barrier for patients awaiting transplantation. In 2013, there were 6256 liver transplantations performed in the USA. That same year, 10,143 patients were added to the waiting list and 3002 either died or became too sick to undergo transplantation [1]. This discrepancy has pushed the transplant community to explore novel ways to increase the donor pool.

Procurement of marginal organs, living donor transplantation and split liver transplantation (SLT) are three innovative ways that the transplant community has tried to meet the expanding demand for more organs. In 1988, the first descriptions of SLT were reported [2, 3]. Initially employed by splitting an adult liver into a left lateral segment for a child and right trisegment allograft for an adult recipient, SLT decreased the pediatric wait list without taking grafts away from the adult recipient population. Despite initial concerns, splitting donor allografts for pediatric and adult patients has been shown to be safe and resourceful [4-7]. Although SLT is a clear solution to the pediatric graft deficit and has decreased wait list times for pediatric recipients [8-10], the role for adult donor pool expansion with full-right-full-left split liver transplantation (FRFLSLT) has only been recently explored.

Just as pediatric-adult SLT was controversial in its infancy; there is concern that FRFLSLT has inferior results when compared to adult/child SLT or whole liver transplant (WLT). Early reports of success with FRFLSLT have been published, however, numbers have been limited [11-17]. These studies are heavily reliant on donor and recipient selection, and investigation into the large scale applicability of FRFLSLT has been lacking. To address the paucity of information available, this study assessed the outcomes of FRFLSLT by comparing full-right (FR), full-left (FL),and whole liver (WL) grafts in adults using United Network for Organ Sharing (UNOS) standard transplant analysis and Standard Transplant Analysis and Research (STAR).

## MATERIAL AND METHODS

The data were collected from the UNOS as reported between 1998 and 2010. Patients with WL, FR, or FL hemiliver allograft were included in the analysis. Pediatric patients and SLT using right trisegment allografts were excluded from the analysis. Means and SDs were given for continuous variables as they were normally distributed, and percentages were provided for categorical variables. To control for multiple pairwise comparisons between graft types, p values from *Student’s t *tests for continuous variables, and χ^2^ for categorical variables, were considered statistically significant at α=0.017 (Bonferroni correction). Patient and graft survival were compared between the graft types using Kaplan-Meier survival analysis. The log-rank test was used to test for equality of survival distributions between the curves for each graft type.

Propensity analysis [18] was conducted to adjust for differences in patient characteristics between liver graft recipients when examining the association between graft type and outcome. A multiple logistic regression model predicting whole graft was used to obtain propensity scores. The model included the covariates: recipient and donor age, MELD score, length of stay (LOS), days waiting for transplant, donor and recipient weight, cold ischemia, donor risk index (DRI), sex, and hepatocellular carcinoma (HCC). The propensity scores (probability of WL) were divided into quintiles to illustrate the similarities in characteristics between the graft types after propensity matching. Patients with extreme scores (0 or >0.97) were excluded from the stratified and adjusted analyses, as it was not possible to match graft recipients by propensity for WL graft at these extreme values. Multivariable Cox proportional hazards models were fit to predict graft failure and mortality. The final adjusted models included only statistically significant predictors (p<0.05) and propensity score. The final graft failure and mortality models included the covariates age of donor, MELD, LOS, and donor weight; and the mortality model also adjusted for recipient age and HCC. All analyses were conducted using SAS 9.2 (SAS Institute Inc., Cary, NC, USA).

## RESULTS

**Figure 1:**
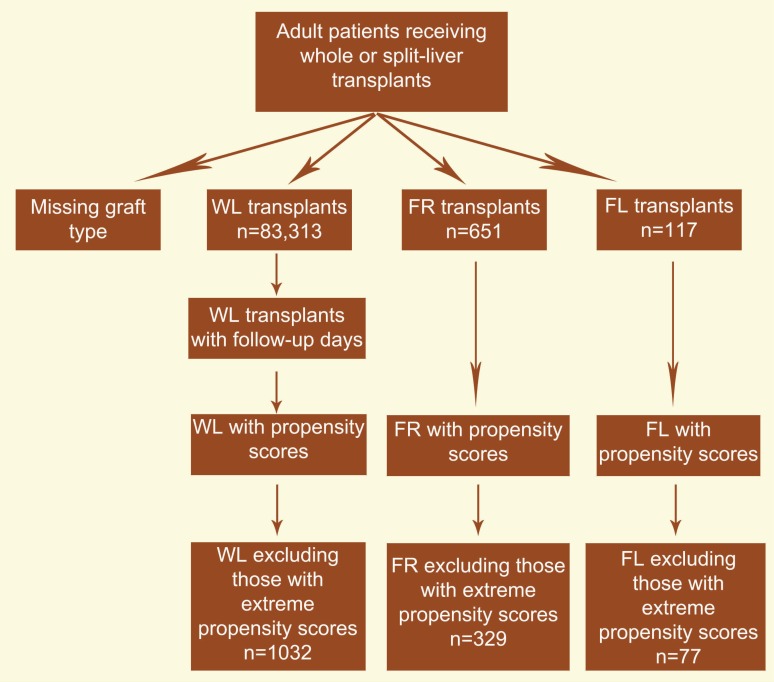
Flowchart of study population

We found that during 1998–2010, a total of 768 adult patients underwent SLT using FR or FL allografts; 651 FR, and 117 FL. At the same time period, 83,313 adult patients received WL allograft (Fig 1). The demographic and clinical characteristics of donors and recipients are shown in Table 1. Donor age was similar in FR and FL while it was significantly higher for WL. Donor weight was higher in the FL and WL groups when compared to FR (77 and 76 *vs*. 72 kg, respectively). Transplant recipient weight was highest for WL patients (82 kg) when compared to FR (72 kg) and FL (69 kg). Those with WL had less time on the liver waiting list than FR and FL (215 *vs*. 344 and 231 days, respectively). FL recipients had shorter cold ischemic time than FR and WL (7.7 *vs*. 8.8 and 8.6 hours, respectively) and were less likely to be male (42% *vs*. 48% and 64%, respectively). MELD scores were similar for FR and FL (18 and 19, respectively) and significantly lower when comparing FR to WL (21). DRI scores were comparable in FR and FL groups (3.9) and significantly higher than WL (2.7).

**Table 1:** Demographics of adult liver transplant patients from 1988–2010 (n=84,081). Figures are either mean (SD) or percent

Variables	WL (1) (n=83,313)	FR (2) (n=651)	FL (3) (n=117)	p value		
				1 *vs*. 2	1 *vs*. 3	2 *vs*. 3
Age (yrs)	51 (11)	52 (11)	51 (12)	0.65	0.74	0.60
Donor age (yrs)	38 (17)	24 (10)	26 (11)	<0.0001	<0.0001	0.17
MELD score	21 (9.2)	18 (8.1)	19 (9.3)	<0.0001	0.12	0.34
Length of stay post-transplantation (days)	21 (43)	19 (22)	21 (19)	0.29	0.98	0.04
Days on liver waiting list	215 (373)	344 (498)	231 (302)	<0.0001	0.65	0.02
Donor weight (kg)	76 (19)	72 (15)	77 (18)	<0.0001	0.36	0.001
Recipient weight at registration (kg)	82 (20)	72 (17)	69 (19)	<0.0001	<0.0001	0.05
Total cold ischemic time (hrs)	8.6 (4.5)	8.8 (4.5)	7.7 (3.7)	0.41	0.05	0.01
DRI	2.7 (0.8)	3.9 (0.8)	3.9 (0.9)	<0.0001	<0.0001	0.31
Follow-up time (days)	1638 (1660)	1471 (1309)	1092 (992)	0.01	0.0004	0.003
Male	64%	48%	42%	<0.0001		
Non-HCC	90%	88%	92%	0.06		
Re-transplantation	8.5%	10%	13%	0.08		

**Figure 2:**
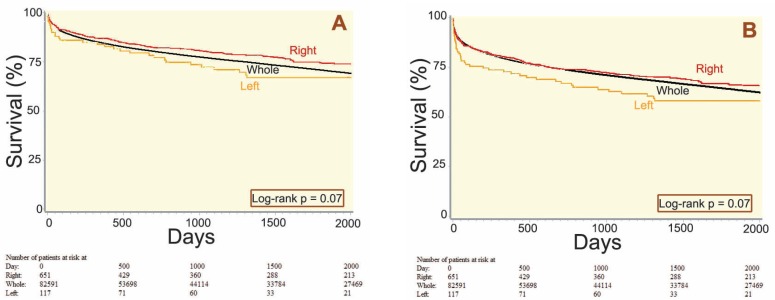
Unadjusted patient (A) and allograft (B) survival after liver transplantation in adults

The unadjusted patient survival of all graft typesis shown in Figure 2A, which suggests a trend toward better allograft survival after FR and WL compared to FL. The 5-year survival rates for FR, WL, and FL were 74%, 71%, and 67%, respectively. However, there was no statistically significant difference between the distribution of survival by graft types (p=0.07). Similarly, when comparing Kaplan-Meier curves between graft types with respect to allograft survival, the FL recipients appear to have the poorest long-term survival with a 5-year survival rate of 58%, while that of FR was 66% and WL was 64% (Fig 2B). The differences between the curves were not statistically significant. 

Propensity scores were obtained for patients using multiple logistic regression predicting WL transplant. The c-statistic for the model was 0.99, which illustrated the model discriminated well. The matched patients were divided into quintiles of propensity for WL graft. After stratifying by propensity quintile, most of the differences in characteristics between recipients of different graft types were no longer statistically significant (Table 2). This allowed analysis of outcomes based on allograft type, thereby reducing selection bias based on donor and recipient characteristics. Propensity adjusted analysis (Tables 3 and 4) showed no significant difference in allograft or patient survival between graft types. Both FR and WL were less likely to have graft failure or death when compared to FL, but the adjusted hazard ratio estimates were not significantly different.

**Table 2:** Characteristics of liver transplant patients both unadjusted and adjusted for propensity quintile (n=1438

	Unadjusted			Quintile 1 (0.005–0.546)			Quintile 2 (0.547–0.850)			Quintile 3(0.851–0.937)			Quintile 4 (0.9373–0.963)			Quintile 5 (0.9631–0.975)		
	WL	FR	FL	WL	FR	FL	WL	FR	FL	WL	FR	FL	WL	FR	FL	WL	FR	FL
	(n=1032)	(n=329)	(n=77)	(n=70)	(n=177)	(n=40)	(n=162)	(n=105)	(n=21)	(n=255)	(n=26)	(n=7)	(n=269)	(n=15)	(n=4)	(n=276)	(n=6)	(n=5)
Mean age	52	52	52	52	52	53	52	51	54	52	52	41*	52	54	44	54	52	55
Mean donor age	28	23*	25	14	21*	21*	26	25	26	26	28	31	31	33	39	30	35	34
Mean MELD	19	18	19	17	18	18	18	18	18	19	20	16	19	17	33	20	21	24
Mean LOS	18	18	20	18	17	16	19	19	24	17	17	21	18	12	30	20	16	18
Mean LOS total	24	21	27	20	19	27	22	25	27	22	20	21	20	17	33	30	19	28
Mean days wait	362	381	263	323	419	277	351	360	326	394	289	204	388	325	77	323	172	124
Mean donor weight (kg)	83	73*	80	55	71*	78*	82	74	83	84	78	87	86	75	87	85	83	70
Mean weight (kg)	76	72*	70*	66	70	72	72	74	67	77	80	72	78	75	71	78	78	64
Mean cold isch. (hrs)	7.7	8.4*	7.2	8.3	8.7	7.5	7.8	7.8	7	7.7	8.1	7	7.6	9.1	5	7.6	8.1	8.1
Mean DRI	3.4	3.9*	3.8*	3.9	4.1*	4*	3.6	3.7	3.6	3.4	3.6	3.5	3.4	3.6	3.6	3.2	3.5	3.6
Male (%)	56**	49	42	37	42	35	51	54	52	60	50	57	65	73	50	52	83	20
Non-HCC (%)	78	81	88	93**	78	93	81	82	76	76	92	86	78	80	100	76	83	100
Re-transplantation (%)	5.6	6.1	12	11**	3.4	13	4.3	8.6	10	8.2	7.7	14	4.1	13	25	4	17.0	0
Infection (%)	16	14	27	15	10	43	29	17	0	9.7	0	50	6.3	50	0	26	0	0

* p<0.017 WL vs. column

** p<0.05 over all groups

**Table 3:** Risk of graft failure (n=1438)

	HR* (95% CI) (WL n=1032, FR n=329, FL n=77)	p value
FR *vs*. FL	0.68 (0.44–1.1)	0.08
WL *vs*. FL	0.74 (0.48–1.1)	0.17

* HR: Hazard ratio

**Table 4:** Risk of mortality (n=1438

	HR* (95% CI) (WL n=1032, FR n=329, FL n=77)	p value
FR *vs*. FL	0.77 (0.46–1.3)	0.32
WL *vs*. FL	0.87 (0.52–1.5)	0.60

* HR: Hazard ratio

## DISCUSSION

SLT for two adult patients usually consists in the generation of a full-right graft (FRG, including segments V–VIII) and a full-left graft (FLG, including segments I–IV) [11-17]. Conversely, conventional SLT produces an extended right graft (ERG, including segments I, IV–VIII) and a left lateral segment (LLS) graft (including segments II and III), which classically benefit one adult and one pediatric recipient [8-10]. Although a few small single-center series have reported acceptable results with FRFLSLT [11-17], the outcomes obtained with this technique are generally worse than with WLT or living-donor liver transplantation [19]. As a result, FRFLSLT is not yet considered a standard procedure [20-23].

We report a series of SLTs for two adult recipients performed at US centers. Although unadjusted results of SLT using FL allografts in adults were inferior to FR, propensity matched analysis showed comparable allograft and patient survival. This experience showed that FRFLSLT can be safely applied in adult patients with acceptable outcomes being anticipated.

The use of propensity scores to create a risk-adjusted, demographically matched cohort based on allograft status (FL *vs*. FR) is an advantage of our study. Propensity scores reduce the entire collection of observed background characteristics to a single variable that appropriately summarizes that characteristics [18]. This step allowed a straight forward analysis of whether the FL or FR groups have enough overlap with respect to observed background covariates, to allow a true assessment of the effect of allograft type status on outcomes. The two groups in our propensity-matched cohort were virtually identical with respect to different background characteristics.

In the face of the severe organ shortage and high waiting list mortality in the adult population, every effort should be made to improve the utilization and outcomes of SLT in adults. If the FRFLSLT procedure is managed correctly, the advantages conferred by a younger donor population, two liver allografts should translate into better allograft and patient survival rates. The negative impacts of both higher recipient MELD scores and prolonged cold ischemic times (CIT) observed in deceased donor WLT are most probably amplified in the SLT group and should be taken into account. SLT represents a potentially underutilized resource in the USA. Factors such as high recipient MELD score and extended CIT may have negative effects on results in SLT and therefore, deter surgeons from performing this procedure. A different allocation system for these grafts that takes into consideration CIT and recipient MELD score should be considered [23].

A limitation of this study is its being a retrospective analysis of UNOS data. We recognize both potential advantages and limitations of such a large national database. The large sample size provides sufficient power to detect meaningful risk factors that may be missed by single-center studies. However, as with any analysis utilizing the UNOS database, our conclusions rely on the assumption that there is no systematic bias generated by reporting error or missing data. The groups are obviously unequal in size and selection criteria for one or the other procedure are not known. However, the primary endpoint for this analysis was allograft and patient survival, which is reliably captured in the UNOS database. Residual or unmeasured confounders that could impact allograft and patient survival include surgical technique and skill level, differences in immunosuppression protocols, the fat content/quality of the allografts, and center-specific practices. Other important determinants of success with SLT such as recipient and donor selection, choice of RL *vs* FL graft, graft weight and quality, surgical details, techniques employed to alleviate small-for-size syndrome, center and surgeon volume/experience were not available in the database. Biliary complications are done well captured in the database either.

Second, we did not have much data on the donor and the quality or size of the allograft. Third, the study did not look at the potential impact of the portal inflow and the hepatic vein outflow modulation in smaller grafts. Fourth, we were not able to analyzed center-specific outcomes. There is a size difference between FR (n=651) and FL (n=117). This would be due to the fact that some of FLs were used in a child or been discarded.

In summary, FRFLSLT in adults can expand the donor pool and provide reasonable outcomes in two adults using FR and FL hemiliver allografts.
